# Forecasting Tunisian type 2 diabetes prevalence to 2027: validation of a simple model

**DOI:** 10.1186/s12889-015-1416-z

**Published:** 2015-02-07

**Authors:** Olfa Saidi, Martin O’Flaherty, Nadia Ben Mansour, Wafa Aissi, Olfa Lassoued, Simon Capewell, Julia A Critchley, Dhafer Malouche, Habiba Ben Romdhane, on behalf of EC FP7 funded MEDCHAMPS project

**Affiliations:** Cardiovascular Epidemiology and Prevention Research Laboratory, Faculty of medicine of Tunis, Tunis, Tunisia; Department of Public Health and Policy, University of Liverpool, Liverpool, UK; Population Health Research Institute, St George’s, University of London, London, UK

**Keywords:** Type 2 diabetes prevalence, Demographic changes, Obesity trends, Smoking trends, Projection, Target strategies, Modeling, Tunisia

## Abstract

**Background:**

Most projections of type 2 diabetes (T2D) prevalence are simply based on demographic change (i.e. ageing). We developed a model to predict future trends in T2D prevalence in Tunisia, explicitly taking into account trends in major risk factors (obesity and smoking). This could improve assessment of policy options for prevention and health service planning.

**Methods:**

The IMPACT T2D model uses a Markov approach to integrate population, obesity and smoking trends to estimate future T2D prevalence. We developed a model for the Tunisian population from 1997 to 2027, and validated the model outputs by comparing with a subsequent T2D prevalence survey conducted in 2005.

**Results:**

The model estimated that the prevalence of T2D among Tunisians aged over 25 years was 12.0% in 1997 (95% confidence intervals 9.6%–14.4%), increasing to 15.1% (12.5%–17.4%) in 2005. Between 1997 and 2005, observed prevalence in men increased from 13.5% to 16.1% and in women from 12.9% to 14.1%. The model forecast for a dramatic rise in prevalence by 2027 (26.6% overall, 28.6% in men and 24.7% in women).

However, if obesity prevalence declined by 20% in the 10 years from 2013, and if smoking decreased by 20% over 10 years from 2009, a 3.3% reduction in T2D prevalence could be achieved in 2027 (2.5% in men and 4.1% in women).

**Conclusions:**

This innovative model provides a reasonably close estimate of T2D prevalence for Tunisia over the 1997–2027 period. Diabetes burden is now a significant public health challenge. Our model predicts that this burden will increase significantly in the next two decades. Tackling obesity, smoking and other T2D risk factors thus needs urgent action. Tunisian decision makers have therefore defined two strategies: obesity reduction and tobacco control. Responses will be evaluated in future population surveys.

**Electronic supplementary material:**

The online version of this article (doi:10.1186/s12889-015-1416-z) contains supplementary material, which is available to authorized users.

## Background

The burden of Non-Communicable Diseases (NCDs), including diabetes and cardiovascular diseases (CVDs), represents a real challenge to health systems. The Eastern Mediterranean Region (EMR) has been recognized as a hotspot for CVDs and type 2 diabetes (T2D). About 47% of the region’s current burden of disease is considered to be due to NCDs, and this may rise to about 60% by the year 2020 according to the Global Burden of Disease project [1].

Diabetes contributes substantially to this burden. The most recent estimates of the burden of diabetes are alarming, suggesting that in 2013 there were 382 million people with diabetes worldwide [2]. Whilst there have been changes in the incidence of type 1 diabetes, it is type 2 diabetes that is largely responsible for the global epidemic of diabetes [3]. Moreover, increasing prevalence of the key risk factors for T2D will also contribute to the urgency of the problem in many parts of the world.

Overweight and obesity, the epicenter of the metabolic syndrome, are the most important risk factors for diabetes [4-6]. Rising rapidly across the world, obesity is now replacing more traditional problems such as malnutrition in developing countries [7]. There is also an extensive body of literature reporting on the association between the incidence of diabetes and active smoking, the leading cause of avoidable death and a prevalent risk factor globally [8-12].

Estimates of the current and future burden of T2D are important in order to allocate community and health resources, and to emphasize the role of life style, and encourage measures to counteract trends for increasing prevalence [13]. In a context of developing countries, such projections are characterized by their scarcity and lack of accuracy: most estimates e.g. those produced by the International Diabetes Federation Diabetes Atlas are based on trends in urbanisation and demographic changes. It seems plausible that predictions based on trends in key risk factors, would be more realistic.

To gain insight on T2D prediction and prevention in Tunisia, we adopted a newly developed tool, the T2D (IMPACT) model, this model was designed for use in settings where available T2D risk factor data might be limited (particularly low and middle income countries), though it was originally developed and validated using data from England and Wales [14].

Recently, the model has been further developed and validated in developing countries such as Saudi Arabia, Palestine, Syria and Turkey [15-19].

This study populates the T2D-IMPACT model with Tunisian data, describes trends in T2D and key risk factors, and uses the model to predict likely future prevalence of T2D according to demographic changes and trends in major risk factors; obesity and smoking. Finally, it uses the model to estimate the likely future impact of different policy scenarios such as policies to improve tobacco control, or reduce obesity.

## Methods

### Model structure

**Figure 1:**
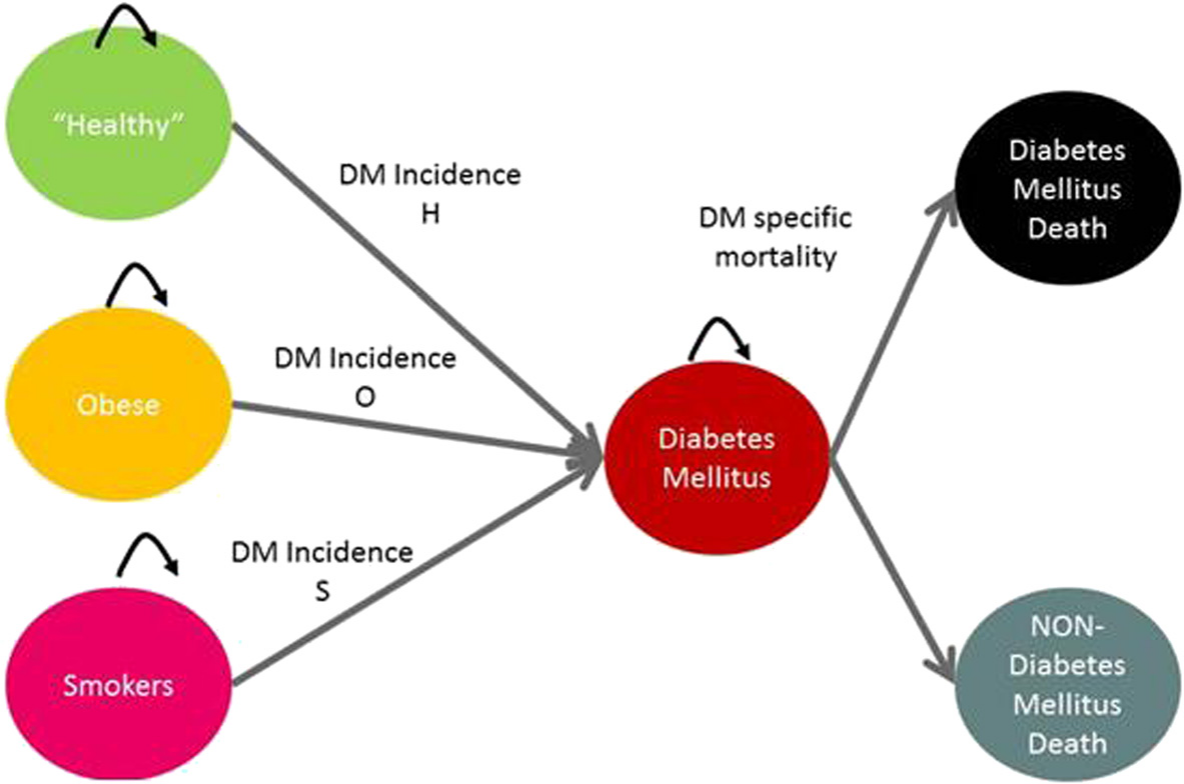
**The type 2 diabetes model structure.**

The total population is divided in several pools: T2D, obese, smoker and “healthy” (i.e.: non obese, non-smokers, no diabetes). A proportion of the population in each pool moves through pathways to other states as described in Figure 1. Population demographic trends are used to inform the relative size of the “starting states”, and transition probabilities are used to estimate the proportion of persons moving from the starting states to the T2D and death states. There are two “absorbing states”: T2D related death and non-T2D related deaths as competing risks for mortality.

Potential overlaps between the healthy, obese and smoking group are managed estimated by calculating the conditional probabilities of membership which allows estimating what proportion of diabetic new cases can be attributed to smoking and obesity at each cycle. These conditional probabilities were obtained from the initial population-based cross sectional survey [16-19].

The model requires a starting T2D prevalence estimate for a given year; in addition to population estimates, and obesity and smoking trends over a time period of interest (all stratified by age group and sex).

A key element of the model is the calculation of the transition probabilities, particularly those related to T2D incidence. For estimating T2D incidence we first estimated a population incidence for T2D based on DM prevalence, population total mortality and T2D relative mortality using freely available epidemiological software, DISMOD [20,21]. T2D relative mortality is calculated using an approach proposed by Barendregt et al. [22,23]. Details of these procedures are available in the Additional file 1.

We developed the model for the Tunisian population aged over 25 years old, for the time period between 1997 and 2027. The data sources for this study include the National Institute of Statistic for annual demographic projections and mortality data [24,25]. Census data are based on the 1994 and 2004 census and include projections of the 2027 population.

The prevalence of T2D in the starting year (1997) was obtained from the Tunisian National Nutrition Survey 1996/97 [26,27]. This survey included a representative sample of 3635 adults over 20 years old. T2D cases were defined as people with measured fasting plasma glucose ≥7 mmol/l or having a previous diagnosis of T2D.

To estimate smoking prevalence in 1997, we used a survey conducted by the National Institute of Public Health in 1996/1997 [28]. This included a representative national sample of 5696 subjects aged 25 and over

For the obesity prevalence in 1997, we used data from the Tunisian National Nutrition Survey 1996/97 [27].

Data in 2005 was obtained from the survey of Transition Epidemiological and Health Impact in the North Africa project (TAHINA), based on a nationally representative sample of 8007 adults aged 35–75 years old. Assumptions were made for the groups aged 25–34 and 75 years and over where prevalence data was not available in 2005 [29] (Table 1).

**Table 1:** **Data sources**

**Parameters**	**Sources**
**Population data**	• National Institute of Statistics in Tunisia
**Mortality data**	
Mortality Causes	• National Public Health Institute
Total Mortality	• National Institute of Statistics in Tunisia [24,25]
**Diabetes**	• Tunisian National Nutrition Survey 1996/97
	• Cardiovascular Epidemiology and Prevention Research Laboratory
**Obesity**	• Tunisian National Nutrition Survey 1996/97
	• Cardiovascular Epidemiology and Prevention Research Laboratory
**Smoking**	• National Institute of Public Health in 1996/1997
	• Cardiovascular Epidemiology and Prevention Research Laboratory
**Relative risk mortality calculation**	Verona Study
**Incidence calculation**	DISMOD

The observed prevalence was presented as prevalence (95% confidence intervals).

### Model validation

Model Validation is an important aspect of any modeling exercise, frequently overlooked. We developed a model for Tunisia, over the period 1997 to 2027, based on prevalence data in 1997. One subsequent survey (TAHINA, described above) with data on T2D prevalence was conducted in 2005, and we therefore compared the model outputs with the observed prevalence estimates for this year [29]. T2D cases in the 2005 survey were defined as people with measured fasting capillary glucose ≥ 6.1 mmol/l or having a previous diagnosis of T2D. For youngest (25–34) and oldest (≥75) age groups (where data was not available from TAHINA), we assumed that the risk factor profile was similar to the adjacent age group [29]. We assumed that fasting plasma glucose ≥7 mmol/l is equivalent to capillary fasting glucose of ≥6.1 mmol/l. This equivalence has recent been demonstrated using Tunisian data [30].

### Type 2 diabetes forecast and policy scenarios

First, we present forecasts for T2D prevalence and the burden up to 2027 (30 years from our initial prevalence estimate in 1997) assuming that current trends in obesity and smoking continue. Obesity trends were capped to avoid implausibly high obesity trends, based on evidence of “leveling off” of obesity which has now been reported in several populations [31,32]. Then, we explored the potential effect of population level interventions to reduce smoking and obesity.

A smoking target was set by the Ministry of Health in 2009 [33]. The smoking strategy is composed of education, sensitization, legislation, tariff policy and coordination with stakeholders to reduce the prevalence of smoking by 20% in 10 years. An obesity target was set by the National Institute of Nutrition in 2010 [34]. The obesity strategy is focused on encouraging the consumption of healthy foods, development of sports activities, dissemination of the information about the importance of healthy nutrition and lifestyle, and early detection of obesity. The target is to reduce the prevalence by 20% in 5 years. However this target appears unachievable so in the model we assumed that the strategy could reduce obesity by 20% over 10 years.

### Sensitivity analysis

We used the analysis of the extremes method [35] consisting of running the model with all parameters set to minimum and maximum realistic values. This is a very conservative approach, but allows a more transparent understanding of the effect of each parameter on uncertainty in model estimates.

## Results

### Demographic and epidemiological changes

Table 2 presents demographic and epidemiological changes in Tunisia in 1997 and 2005.

**Table 2:** **Tunisian population aged 25 years and above, obesity and smoking prevalence trends by gender and age groups in 1997 and 2005**

	**1997**								**2005**							
**Age groups**	**Population N (Thousand)**		**Diabetes mellitus type II (%)**		**Smoking (%)**		**Obesity (%)**		**Population N (Thousand)**		**Diabetes mellitus type II (%)**		**Smoking (%)**		**Obesity (%)**	
	M*	W**	**M**	**W**	**M**	**W**	**M**	**W**	**M**	**W**	**M**	**W**	**M**	**W**	**M**	**W**
25-34	733	751	8.0	6.8	58.8	0.9	4.9	17.3	798	849	13.9	9.2	50.0	6.8	7.3	17.3
35-44	549	546	8.6	8.6	49.7	1.9	11.3	28.8	671	706	15.9	11.8	53.8	2.6	14.4	33.7
45-54	318	329	15.6	13.6	43.4	2.0	8.3	38.8	522	516	17.2	14.8	47.0	2.0	20.4	45.5
55-64	261	264	18.4	21.0	33.4	2.0	7.4	32.5	282	299	22.6	21.9	42.4	2.0	17.0	35.2
65-74	174	201	15.3	22.2	33.4	1.4	8.2	25.9	226	225	16.9	21.3	26.2	2.2	10.4	28.1
75+	90	85	23.5	26.4	33.6	0.9	10.0	15.7	126	119	18.7	16.8	18.8	1.4	10.0	19.9
Total	**2125**	**2175**	**13.5**	**12.9**	**48.0**	**1.5**	**7.9**	**26.0**	**2625**	**2713**	**16.1**	**14.1**	**45.0**	**2.5**	**13.1**	**29.9**

*M: Men.

**W: Women.

**N: Number.**

#### Demographic changes

Tunisia has about 10 million inhabitants. We considered the population aged over than 25 years. For this age group, the number of men is estimated to grow from 2125 thousand in 1997 to 3879 thousand in 2027 and from 2175 to 4117 thousand for women. The proportion of young people aged 25–34 years will decrease from 34.5% in 1997 to 22.9% in 2027. By 2027, 16% of the population 25 years and above will be aged over 65 compared with 13% in 1997.

### Observed prevalence of type 2 diabetes

The observed prevalence of T2D in Tunisia in 1997 was overall 13.1% (confidence interval (CI):11.9-14.3); 13.5% (CI: 11.5–15.4) in men and 12.9% (CI: 11.4–14.3) in women, with a clear tendency to increase with age, except for men aged between 65 and 74 years.

### Obesity and smoking trends

Obesity prevalence rose from 7.9% in 1997 to 13.0% in 2005 in men (annual increase of 8%) and from 25.9% to 29.9% in women (annual increase 2%). Based on our assumptions, obesity prevalence would increase to 27.0% in men and 44.0% in women by 2027.

The annual smoking trends showed a decrease in men (−1%) (from 48% to 45%), but an increase in women. The annual percentage increase among women was 9% (from 1.5% to 2.5% respectively between 1997 to 2005). The projected prevalence for 2027 is 47.2% in men and 7.8% in women.

### Type 2 diabetes p

**Figure 2:**
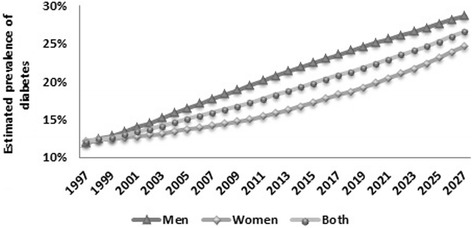
**Estimated prevalence of type 2 diabetes.**

Assuming these annual trends in risk factors continue, the forecast prevalence of T2D for 2027 is overall 26.6% (min 22.8 –max 29.6): 28.6% in men (min 24.6 –max 31.6) and 24.7% in women (min 21.2 –max 27.8) (Figure 2, Table 3). The total number of Tunisian people with T2D is projected to rise from 516 thousands in 1997 to 2125 thousands in 2027, an increase of 300%. This huge rise of the T2D prevalence is mainly due to the increase of young people aged 25–54 years with T2D.

**Table 3:** **Scenario projection rate of prevalence of type 2 diabetes (T2D) by gender with sensitivity analysis (Minimum-Maximum)**

	**Men (%)**	**Women (%)**	**Total (%)**
2015	22.3 (18.7-25.0)	17.0 (14.7-19.2)	19.6 (16.6-22.0)
2020	24.1 (20.6-26.6)	18.6 (16.2-20.6)	21.3 (18.4-23.5)
2027	26.1 (23.0-27.7)	20.6 (18.6-22.2)	23.2 (20.7-24.9)

The type 2 diabetes model structure.

### Scenario projections

If the national strategies on tobacco and obesity control achieve their objectives a 3.3% (min 2.1- max 4.6) reduction in T2D prevalence would be achieved by 2027; 2.5% (min 1.5-max 3.9) in men and 4.1% (min 2.7–5.6 max in women) (Table 3), this corresponds to 266,691 (min 135,040- max 458,418) fewer persons with T2D (98,479 fewer men and 168,223 fewer women). Detailed results with sensitivity analysis are shown in Table 3.

### Validation

**Figure 3:**
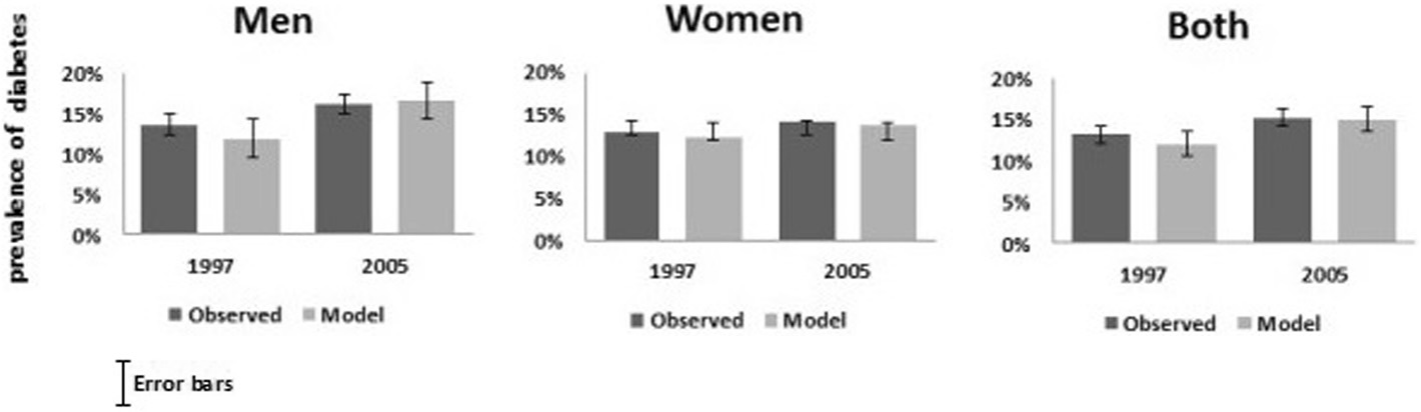
**Comparison of model and observed estimates for type 2 diabetes prevalence by gender: Tunisia 1997 and 2005.**

For validation, comparisons of the model estimates showed a close fit with the observed prevalence of T2D in Tunisia in the TAHINA survey in 2005 (the only point available for validation): the model prevalence estimate was 15.1% overall (observed 15.2%), 16.2% (16.1%) in men and 14.0% (14.1%) in women. The modeled and observed estimates of T2D prevalence are shown in Figure 3.

## Discussion

The forecast prevalence of T2D for 2027 is 26.6% overall (28.6% in men and 24.7% in women) with a 300% increase in the number of persons with T2D between 1997 and 2027. Two initiatives for primary prevention of obesity and smoking have been proposed. If these strategies are implemented effectively, a 3.3% reduction in T2D prevalence could be achieved by 2027 (2.5% in men and 4.1% in women) and will result in 13% fewer people with T2D by 2027.

These T2D projections are somewhat higher then predictions made for developing countries by the International Diabetes Federation (IDF) in 2011 for the year 2030 [36]. The likeliest explanation for this difference is that the IDF projections were based only on demographic changes and urbanization, ignoring changes in population risk factor profiles, other than what may be implicit via urbanization. In fact, recent trends in T2D risk factors rates and major advances in the understanding of the natural history of T2D have not been formally incorporated into prior forecasts of the burden of T2D for the Tunisian population.

In developing our forecasting model, we model two dynamic processes to estimate the T2D population. First, new people are diagnosed and added to the T2D population. Second, other individuals with existing T2D die and leave this sub-population. With the balance of these two processes, the prevalence of T2D in the total population changes on an annual basis, depending on factors such as the rate of obesity, active smoking and age of those at risk.

The model provides a rigorous assessment of the future burden of T2D that accounts for both the natural history of the disease and demographic changes. More importantly, the model can also be used to provide estimates of the impact of alternative policy strategies tackling obesity and smoking. The current obesity trends, when projected into the future result in substantial increases in this main determinant of T2D. The rate of increase is more pronounced among men (annual increase of 6% versus 2% in women). Similarly, smoking rates remain high among men and are lower but increasing in women. If these rates continue to 2027 nearly half of all men and 8% of women will be current smokers.

Obesity is a global epidemic, and rates are particularly high in the Middle East (38–44%) compared with 21% in USA and 23% in Canada [37]. In developing countries such as Palestine diabetes mellitus prevalence estimated by the model forecasts were 20.8% for 2020 and 23.4% for 2030 [17]. A recent study in Saudi Arabia was published to validate the Markov model, this found that prevalence estimates from this new Markov model were consistent with the 2005 national survey and very similar to the Global Burden of Diseases (GBD) study [16].

In Tunisia, adding urgency to the T2D debate is the finding that the number and proportion of obese people is dramatically increasing. Our model predicts that 21.1% of men and 40.5% of women will be obese in 2027, increasing from 6.8% and 22.6% respectively at the time of the 1997 nutritional survey [36]. Whilst these predicted obesity prevalence estimates may seem very high, they are in line with the alarming findings of a more recent survey conducted in 2005 in the great Tunis area (the capital of Tunisia). Whilst obesity is more common in urban areas [26] this survey estimated that 34% of adults (and 46% of women were obese) [38].

Recent studies among adolescents have shown similar dramatic increases. Skhiri et al. found that overweight had increased markedly among those aged 15 to 19 years from 2.9% in 1997 to 17.4% in 2005 for boys and from 13.5% to 20.7% for girls for the same period. Furthermore, abdominal obesity appeared as a significant feature in most overweight adolescents. These findings are very alarming since these adolescents are at high risk of developing later chronic diseases [39].

Smoking is also globally recognized as the most serious public health problem. As predicted by the model, female smoking prevalence will increase from 2.53% (TAHINA survey 2005) to 7.8% by 2027. Although tobacco consumption seems to have fallen slightly in men during these last 20 years, it is still at a very high level and the consequences of this tobacco use in term of mortality will be considerable [40]. We estimate that 47.2% of men will smoke in 2027 increasing from 45.8% in 2005. Comprehensive tobacco control policies including multiple and coordinated actions to prevent the uptake of smoking in young people, and help smokers stop smoking are urgently needed.

### Limitations of the study

The modeling approach used in the study synthesized the key risk factors to help predict T2D prevalence in the future. It also provided estimates of the impact of alternative policy scenarios. Additionally, the model assessed the potential maximum and minimum plausible effects of these factors using rigorous sensitivity analyses which examined systematically the influence of uncertainties in the assumptions used in the studies.

This modeling approach also has obvious limitations. The model assumes “non-reversibility”: individuals cannot move from “healthy” to obese and back to “healthy” again, or from smoker and back to “healthy” or the like. This could theoretically overestimate obesity and smoking, and thus T2D prevalence. However, in Tunisia available evidence suggests that very few smokers have ever quit and that people who are overweight or obese rarely become a healthy weight again. Thus, this limitation may not be significant in this population [28]. The model was also based on many assumptions that are reasonable but still uncertain. The underlying assumption in these estimates is that a person with T2D of a particular age will have the same risk of dying in 2027, as they did in 1991 (date of the VERONA study) [23].and a person who is obese or a smoker will have the same risk of developing T2D in 2027, as he or she had 20 years previously. Nevertheless, since most studies indicate rising risk and decreasing mortality over time, these estimates are likely to be conservative. Furthermore, an important assumption is that this method requires a population in equilibrium, since the consistency between epidemiological estimates depends on the underlying trends in each parameter. However it is difficult to disentangle these effects from data inaccuracy. The robustness of the approach to violations of these assumptions is not known. We estimated diabetes incidence using a previously published and validated tool (DISMOD2) but it is notoriously difficult to assess the accuracy of these estimates, given the limitations (reversibility and changes in dynamic equilibrium) mentioned above.

Moreover, a large number of factors that increase T2D risk were not considered in our model, such as body fat distribution, duration of obesity, weight gain, physical activity, diet, the in utero environment, infant feeding practices, childhood stunting and genetic factors [41].

For smoking, the lack of adjustment for socioeconomic status in the meta-analysis (only 6 studies adjusted for socioeconomic status or education), diet (only 2 studies), physical activity (only 13 studies), and alcohol consumption (only 14 studies) could inflate the association between smoking and T2D.

Data used were obtained from various surveys, methodologies and sampling strategies as detailed in the Additional file 1. The demographic information was obtained from the census data and the risk factor trends were obtained from well-designed epidemiological studies and surveys using the WHO STEPS methodology.

Finally, certain assumptions were needed to fill in the gaps for missing information. Assumptions were thus made for the groups aged 25–34 and 75 years and over where prevalence data was not available. These assumptions are transparent, being systematically detailed in the Additional file 1, supported by local expert opinion and literature from the region and included in the sensitivity analysis.

### Prevention of diabetes

In Tunisia, the importance of diabetes and its impact was well recognized early in the 1990s. Organizationally, national programs for hypertension and diabetes were developed. These programs are focusing on capacity health personal training, early detection and treatment and patient education. Obesity and tobacco control strategies have been developed [42]. Obesity reduction is focusing on physical activities and healthy diet promotion, while tobacco control is based on legislation, education at work sites and schools and the implementation of outpatient smoking cessation clinics in health centers. These came together in a 2008–2013 Action Plan on NCD Prevention and Control. Reducing risk factors or arresting the rise in disease levels will depend on adopting high-impact measures at the population level, coupled with active engagement of relevant health sectors. However, it is commonly acknowledged that there is a gap between the recommendations and practice.

Our results suggest that a population primary prevention strategy would result in a considerable reduction of the diabetes burden, but it will probably need to be more ambitious in its targets.

Relatively small improvements in nutrition, reductions in obesity and smoking and increases in physical activity, if applied across a whole population, can have a large impact on the rates of T2D, and other chronic diseases that share the same risk factors (such as cardiovascular disease and many cancers). Much more attention needs to be focused on how to achieve such population-wide changes. With this in mind, the International Diabetes Federation (IDF) population strategy requires the governments of all countries to develop and implement a National Diabetes Prevention Plan. This national plan would encompass many groups including schools, communities (for example, religious and ethnic groups), industry (marketing, investment policy, product development) and the workplace (health promotion within the working environment) [43].

In Finland, one of the first countries to implement a national strategy for primary prevention of T2D, the prevention and care of people with T2D are taken very seriously. The DEHKO project in Finland provides an example of a comprehensive approach to T2D prevention and management, which aims to improve nutrition and physical activity across the population, identify and provide individualized support to those at high risk of T2D and assist with the early detection and management of those who are already have T2D [44].

Thus far, the interim evaluation reports of DEHKO show that the objectives being met, some ahead of schedule. Preventive action has gained a firm foothold in the primary care sector, and diabetes education is increasing. The Finnish government is a model for nations facing the growing need for diabetes prevention and care, and the Finnish Diabetes Association, a nongovernmental organization, has shown creativity and innovation in the delivery of programs for diabetes in the country [45].

## Conclusions

This model provides a reasonably close estimate of diabetes prevalence for Tunisia over the 1997–2027 period, compared with an independent prevalence survey in the same population. Diabetes burden is now a significant public health challenge, and our model predicts that its burden will increase significantly in the next two decades. Tackling obesity and other diabetes risk factors therefore needs urgent action and a review of the current national strategy targets [46].

### Ethical approval

None required as secondary analysis of publicly available data.

## Additional file

Additional file 1:
**Technical appendix.**
