# Supplementary material for: Forecasting Tunisian type 2 diabetes prevalence to 2027: validation of a simple model
Source: BMC Public Health. 2015 Feb 7;15:104. doi: 10.1186/s12889-015-1416-z (PMC4348374; doi:10.1186/s12889-015-1416-z)
Supplement: Additional file 1: — Technical appendix. [file 12889_2015_1416_MOESM1_ESM.pdf]

The IMPACT diabetes Model for Tunisia

Technical Appendix for the Baseline Model

Olfa Saidi

Martin O'Flaherty

Nadia Ben Mansour

Wafa Aissi

Olfa lassoued

Simon Capewell

Julia Critchley

Dhafer Malouche

Habiba Ben Romdhan

On behalf of the MEDCHAMPS Project

January /2015

Contents

1. The Model 3
Methods Overview 3
The Model Structure: 3
The Model Workbook 4
Sensitivity Analysis: 5
2. Data needs 5
Minimum dataset 5
Deriving model parameters 6
3. Model Validation 10
Background and aims: 10
4. Data Sources 11

1. The Model

The purpose of the MEDCHAMPS IMPACT Diabetes model is to provide estimates of future diabetes prevalence and offer a modeling platform for policy decision making.

We estimated the overlaps using the following approach: First, we estimated the conditional probability of being a smoker and obese, and assigned those individuals to the smoker group. Second, we estimated the proportion of diabetes persons among the obese population using a population attributable risk approach and discount them from the obese pool. Finally the healthy group was calculated as the population – obese –smoker-diabetes pools.

Methods Overview

The model integrates information on population, obesity and smoking trends at a given point in time to estimate diabetes prevalence in the future.

The population is partitioned in three states (healthy, obese and smokers) and from them, number of diabetes patients and diabetic and non-diabetic deaths are estimated for subsequent time periods using a Markov approach.

The effect of policy decisions can be modelled by the estimated effect on risk factors trends, and the trend parameter can be modified to model increasing, decreasing or stable trends in the prevalence of obesity and or smoking. Another way of exploring policy options (like, for example, multifactorial lifestyle diabetes prevention interventions) can be modelled through the modifications of the diabetes incidence parameter.

The Model Structure

Models are simplifications of reality. In order to keep the model simple but at the same time useful, many compromises on the way the disease epidemiology is modelled are necessary. More complex models usually require different approaches and an amount of data that probably will not be available for the participating countries. A graphical description of the model is presented in figure 1 in the text.

We assume that the population can be divided in several pools: Diabetes mellitus, Obese, Smoker and "healthy" (eg: non obese, non smokers, non diabetics). A proportion of the population in each pool moves through pathways to other states as described in figure 1. Population demographic trends are used to inform the relative size of the "starting states", and transition probabilities are used to estimate the proportion of persons moving from the starting states to the diabetes and death states. There are two "absorbing states": Diabetes Mellitus (DM) related death and Non DM related deaths. In this way, mortality competing

risks are modelled. Potential overlaps between the healthy, obese and smoking group are managed by calculating the conditional probabilities of membership.

The Model Workbook

The workbook is a MS Excel spreadsheet, structured in tabs. These tabs serve different purposes, but for the end-user the key tabs are the Data Input, Dashboard and Validation tabs.

The following is a more detailed description of each tab and its purpose, with a more thorough description of the key ones.

The Data Input tab:

The following sections are:

Population data: used to input the age and gender structure of the population being modelled, and the populations projections

Morbidity data: cross sectional data on diabetes prevalence, obesity and smoking is provided. Trend parameters can be set up here also or use the defaults. Currently, linear trends can be modelled, but any other type of trend can be implemented.

The Dashboard tab:
Figures and tables presenting the model estimated diabetes prevalence are presented here.

The validation tab:

This tab summarizes important validation information for the country, if the validation exercise has been conducted. .

Outputs:

The raw outputs of the model and the sensitivity analysis is stored here. Available information: numbers of diabetes patients, diabetes prevalence, minimum and maximum estimates.

SA layer:

Calculations for estimating transition probabilities are performed here. ***Markov Chains:***

They are implemented in separate tabs for each gender and age group. Two sets of chains are implemented here for the baseline and scenario runs.

Sensitivity Analysis:

We used the analysis of the extremes method (Briggs), consisting in running the model with all parameters set to a minimum and maximum realistic values. This is a very conservative approach, but allows a more transparent understanding of the weight of each parameter regarding model outcomes. The Sensitivity Analysis is updated by running a macro.

2. Data needs

Minimum dataset

The model requires data by 10 year age and gender bands, starting at 25, ending 75+. Details on the sources are available in the table in section 4. The actual values used in the model are presented in section 5.

1. Initial year:
   - 1.1. Population
   - 1.2. Diabetes prevalence
   - 1.3. Obesity prevalence
   - 1.4. Smoking prevalence (current smokers)
   - 1.5. Total mortality (needed for DISMOD
   - 1.6. based estimation of incidence rate)
2. Subsequent years for validation purposes
   - 2.1. Population
   - 2.2. Diabetes prevalence (as many time points as possible, particularly the latest available year)
   - 2.3. Obesity prevalence (as many time points as possible)
   - 2.4. Smoking prevalence (as many time points as possible)
3. Subsequent year for forecasting purposes
   - 3.1. Population projections
   - 3.2. Obesity trends (assumption, initially a assuming a linear increase per year will be useful. We can also extrapolate from the existing trend data)
   - 3.3. Smoking trends (assumption, initially a assuming a linear increase per year will be useful. We can also extrapolate from the existing trend data)
4. DISMOD INPUTS:
   - 4.1. Incidence
   - 4.2. Case Fatality
   - 4.3. Mortality

Details of the data used are found in the data sources section (Section 4).

Deriving model parameters

One of the key aims of the model is to use the minimum data requirements possible. We adapted the methods developed by Barendregt et al (Epidemiology Volume 11(3), May 2000, pp 274-279) to estimate two of the key parameters.

Diabetes incidence and specific mortality

The MEDCHAMPS Diabetes Markov model use diabetes mellitus incidence and mortality as one of the critical data inputs that need to be provided by the participants countries, to help localize and calibrate the model to each population.

Since reliable and country specific sources of incidence data are probably not available, an estimate of it is needed.

We adapted a method to estimate it, and provide as an example, the estimation of baseline diabetes incidence for **Tunisia**.

The method

Incidence, mortality and prevalence are closely related to each other, in a way that only some values for each parameter are consistent with the other parameters at a given time. This property has been used by Barendregt et al to estimate diabetes mellitus incidence in the Netherlands (Epidemiology Volume 11(3), May 2000, pp 274-279).

The technique use as input whatever parameters that are known and using a multistate generic disease model using a lifetable markov approach, estimate revised parameters for the inputed ones and estimates for those unknown. This method has been implemented in software called DISMOD II (http://www.who.int/healthinfo/global_burden_disease/tools_software/en/)

For the MEDCHAMPS project, it is expected that the only available parameter is probably diabetes mellitus prevalence (either self reported or using ADA/NHANES definitions). However, diabetes excess mortality can be estimated from total mortality data (See Barendregt) using literature based estimates of mortality relative risk and disease prevalence, and we can safely assume that the remission rate for diabetes in effectively 0. Thus, the only parameters needed (by age and gender) are diabetes mellitus prevalence, population structure and population general mortality.

An important assumption is that this method requires a population in equilibrium, since the consistency between epidemiological estimates depends on the underlying trends in each

parameter. However it is difficult to disentangle these effects from data inaccuracy. The robustness of the approach to violations of these assumptions is not known.

This method produces a “population incidence”, eg, the incidence both for exposed and unexposed people to diabetes risk factors.

However, the MEDCHAMPS diabetes model needs incidence in the non exposed, since incidence for obese persons and smokers is derived from that baseline incidence by using literature based relative risks.

It has been proposed that the incidence of a disease in a population is a weighted sum of the incidence among the exposed and the incidence among the unexposed to a risk factor(Epidemiology By Moyses Szklo, F. Javier Nieto, equation 3.8 in page 101) (equation 1).

(Equation 1)

$$i_p = i_e \times p + i_u \times (1 - p) \mathrm{i_p} = \mathrm{i_e} \times \mathrm{p} + \mathrm{i_u} \times (1 - \mathrm{p}) \ ,$$

Where i_p is the population incidence, i_e is the incidence amongst the exposed, i_u is the incidence amongst the unexposed and p is risk factor prevalence.

Since the incidence in the exposed is the incidence in the unexposed times the relative risk (RR) (Equation 2),

(Equation 2)

$$i_e = RR \times i_u \ ,$$

it is possible to derive from this two ideas the value for the unexposed incidence from the incidence in the population. Replacing equation 2 in equation 1

(Equation 3)

$$i_p = RR \times i_u \times p + i_u \times (1 - p)$$

And then extracting I_u (Equation 4)

(Equation 4)

$$i_u = \frac{i_p}{(p \times RR - p) + 1}$$

Estimation of the incidence, case fatality and mortality parameters for Tunisia, 1997

This section describes the method used to estimate diabetes mellitus type II incidence for the Tunisian population in 1997.

DISMOD need at least 3 inputs. For this case, we used diabetes mellitus prevalence, diabetes mellitus remission rate and diabetes mellitus relative risk for mortality.

Diabetes prevalence was obtained from ***Tunisian National Nutrition Survey 1996/97***

Tunisian National Nutrition Survey 1996/97: The survey was cross-sectional from June 1996 to December 1997 on a nationally representative sample (5669, 200 inhabitants in 1995): 1735 households with a total of 5815 adults over 20 years old.

- ✓ Definition: Type 2 diabetes cases were defined as subjects with measured Fast Plasma Glucose **>=7 mmol/l** or having a previously diagnosed diabetes.

We can safely assume that diabetes mellitus remission rate is 0, and diabetes mellitus relative risk for mortality can be estimated as proposed by Barendregt et al, based in the usual RR for mortality (mortality in diseased/mortality in non diseased) and disease prevalence. The formula is

(Equation 5)

$$RR_{ADJ} = \frac{RR}{pRR + 1 - p}$$

Where RR adj is the relative risk mortality, RR is the usual relative risk for mortality (mortality diseased/mortality healthy) and p is disease prevalence. The Verona Study (Ref: Muggeo M, Verlato G, Bonora E, et al: The Verona diabetes study: a population-based survey on known diabetes mellitus prevalence and 5-year all-cause mortality. Diabetologia 1995, 38(3):318–325) provides age and gender specific values for RR. A summary of the calculations for this parameter is presented in table1.

Table 1. Estimating RRadj

Age	Year	Verona RR	DM Prevalence 1997	RRadj
**25-34**	men	2.33	1.05%	2.30
	women	3.43	1.05%	3.34
**35-44**	men	2.33	2.40%	2.26
	women	3.43	1.35%	3.32
**45-54**	men	2.33	4.35%	2.20
	women	3.43	2.40%	3.24
**55-64**	men	2.13	8.70%	1.94
	women	2.33	4.65%	2.19
**65-74**	men	1.5	10.50%	1.43
	women	2.27	9.90%	2.02
**75+**	men	1.13	13.05%	1.11
	women	1.32	9.90%	1.28

Table 2. DISMOD Calculations

DISMOD RUN:			
DISMOD RUN Parameter setup	Trend 9% men, 3% women based on diabetes trends, No IRAN, 2004 prevalence		
Prevalence source: 2004		Weights:	P1, RR 0.5, R1
Remission 0		Trend:	10 y3% men and women, assumed
RR mortality		Input adjustments:	Sigmoid, manual adjust, smooht
Incidence (intput) NO			

Incidence			Case Fatality Rate			Mortality		
**25-34**	0,0082	0,0053	**25-34**	0,0021	0,0014	**25-34**	0,0001	0,0001
**35-44**	0,0123	0,009	**35-44**	0,0034	0,0033	**35-44**	0,0004	0,0003
**45-54**	0,0146	0,0124	**45-54**	0,0061	0,0063	**45-54**	0,001	0,0009
**55-64**	0,0147	0,013	**55-64**	0,0106	0,0082	**55-64**	0,0021	0,0016
**65-74**	0,0135	0,0135	**65-74**	0,0141	0,0182	**65-74**	0,0031	0,0043
**75+**	0,013	0,0202	**75+**	0,0225	0,0474	**75+**	0,0052	0,0125

3. Model Validation

Background and aims:

Model Validation is an important aspect of any modeling exercise, frequently overlooked.

We developed a model for **Tunisia,** over the period **1997 to 2027.**

During that period, subsequent surveys were conducted in **1997, 2005 and 2009** and we compared the model outputs with the **Tunisian National Survey 2005 (TAHINA):** The survey was cross-sectional from April to September 2005 and the target population was all 35-70 year old of both genders. With a total of 8007 subjects. It was based on a nationally representative stratified two-stage cluster sample of households according to the seven administrative regions of Tunisia.

4. Data Sources

Table 3. Data inputs

Data Item	Source	Comments
	**1. Initial year:1997**	
1.1. Population	**National Institute of Statistic :** **Ref 24:** National Institute of Statistics, Tunisia. Yearly Statistics Report: N° 40; 1997.	**Excellent Data**
1.2. Diabetes prevalence	**Tunisian National Nutrition Survey 1996/97** **Ref 27:** Institut national de nutrition et de technologie alimentaire. Evolution de l'état nutritionnel de la population tunisienne. Enquête nationale 1996-1997.	**Tunisian National Nutrition Survey 1996/97: The survey was cross-sectional from June 1996 to December 1997 on a nationally representative sample: 1735 households with a total of 3635 adults over 20 years old.**
1.3. Obesity prevalence		
1.4. Smoking prevalence (current smokers)	**Tunisian National survey, INSP 1996-1997** **Ref 28 :** Institut National de Santé Publique. Epidémiologie des broncho-pneumopathies chroniques chez l'adulte. Résultats de l'enquête nationale menée en 1996-1997.	**Tunisian National survey, INSP 1996-1997: This study was conducted in 1996 of a representative national sample of 5696 subjects aged 25 and over. Data were collected by means of a questionnaire in Arabic.**
1.5. Total mortality (needed for DISMOD based estimation of incidence rate)	**National Institute of Statistic** **Ref 25:** National Institute of Statistics, Tunisia .Yearly StatisticsReport: N° 52; 2009	**Excellent Data**
	**2. Subsequent years for validation and forecasting purposes**	
2.1. Population trends	**National Institute of Statistic** http://www.ins.nat.tn/indexfr.php	**-For women aged 65-74 year- old, linear extrapolation is used between 1997 to 2004 and 2012 to 2017**
2.2. Diabetes trend	**Tunisian National Survey 2005(TAHINA)** **Ref 29 :** Epidemiological Transition and Health Impact in North Africa. Cardiovascular Epidemiology and Prevention Research Laboratory. TAHINA Project Final Report, 2006.	**-The population was all 35-70 year-old of both genders: For older age groups, we assumed that the risk factor profile is similar to the previous older age groups. For obesity, we assumed that the age group 25-34 is 1/3 of 35-44 (Experts opinions)**
2.3. Obesity trends		
2.4. Smoking trends		
